# Undilated versus dilated monoscopic smartphone-based fundus photography for optic nerve head evaluation

**DOI:** 10.1038/s41598-018-28585-6

**Published:** 2018-07-06

**Authors:** Maximilian W. M. Wintergerst, Christian K. Brinkmann, Frank G. Holz, Robert P. Finger

**Affiliations:** Department of Ophthalmology, University of Bonn, Ernst-Abbe-Str. 2, 53127 Bonn, Germany

## Abstract

Smartphone-based fundus photography (SBFP) allows for a cheap and mobile fundus examination with the potential to revolutionize eye care especially in low income settings. The purpose of this study was to assess the impact of pupil dilation on image quality in optic nerve head (ONH) imaging and vertical cup-to-disc ratio (vCDR) evaluation with SBFP. Eyes with glaucoma or suspected to have glaucoma were imaged with conventional digital fundus photography (CFP) and SBFP undilated and following dilation, all monoscopically. SBFP was possible in 74% of eyes without dilation and in 98% following dilation. Better image quality on SBFP was achieved with dilation and complete visualization of the optic disc rim was possible in 46% of images without dilation and on 94% of images with dilation. VCDR measurements on images obtained following dilation highly correlated with measurements on CFP (coefficient of correlation r = 0.91, p < 0.001), whereas vCDR on images obtained without dilation correlated less well with CFP (r = 0.70, p < 0.001). SBFP for ONH evaluation is promising, however dilation appears mandatory to achieve results comparable to optic disc evaluation on CFP. ONH imaging with smartphones without dilation might bear the risk of underestimating the CDR and hence overlooking patients at risk for glaucoma.

## Introduction

Smartphone-based fundus photography (SBFP) allows for a cheap and mobile fundus examination and documentation with the potential to revolutionize eye care especially in low income countries^1–4^. With the advent of SBFP multiple applications in ophthalmology have been accomplished including smartphone-based diabetic retinopathy screening^5–10^. Although optic nerve head (ONH) evaluation would be another natural application of SBFP, there is a dearth of literature on the application of SBFP for glaucoma screening to date^11,12^. As the vertical cup-to-disc ratio (vCDR) has proved to be a simple, relatively robust index of glaucomatous loss of the neuroretinal rim^13,14^, ONH evaluation using SBFP may be applicable in glaucoma screening. If the camera’s light beam and the illumination source are adequately coaxial, SBFP can even be performed without pupil dilation, which further simplifies its application^15,16^.

In the study by Russo *et al*. undilated SBFP with a D-Eye adapter and an iPhone 5 s was compared to undilated clinical 90D lens biomicroscopy for vCDR evaluation^11^. Agreement between the two modalities was good (kappa = 0.63) and SBFP was possible in 97% of the eyes (104 out of 107). However only undilated SBFP has been performed. Bastawrous and coworkers compared dilated SBFP with a Peek Retina adapter and a Samsung S3 to dilated conventional digital fundus photography for vCDR evaluation^12^. Also this study revealed good agreement (kappa = 0.69) and SBFP was possible in 80% of the eyes (2322 out of 2920).

With glaucoma being a major cause for irreversible blindness more studies on the applicability of SBFP for glaucoma screening and the evaluation of glaucomatous optic discs are warranted. The comparison of dilated to undilated SBFP with the latter being more feasible in a low income setting is of particular interest. So far, image quality and agreement in vCDR evaluation have not been compared between undilated and dilated SBFP. Thus we performed this study.

## Methods

### Subject recruitment

Patients were consecutively recruited from the glaucoma outpatient clinic at the Department of Ophthalmology of the University of Bonn, Germany. Ethical approval was obtained from the ethics committee of the University of Bonn and informed consent was obtained from all study participants prior to study inclusion. The Declaration of Helsinki was followed. Exclusion criteria were any retinal diseases and severe media opacities.

### Image acquisition

**Figure 1:**
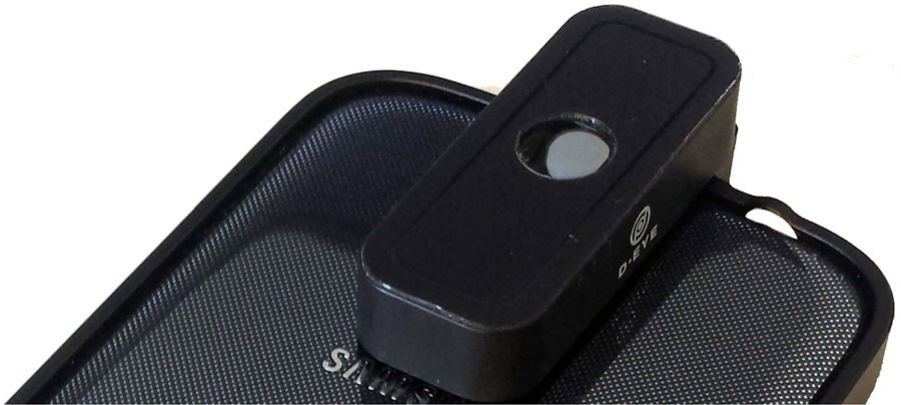
Galaxy S4 equipped with the D-Eye adapter. The D-Eye adapter is magnetically attached to a frame that encompasses the Galaxy S4.

Eyes were imaged with a Galaxy S4 (Samsung Electronics, Seoul, South Korea) using the D-Eye adapter (Fig. 1, version from 2016, D-EYE S.r.l., Padova, Italy) monoscopically first undilated and then dilated. The D-Eye adapter’s optics consist of a negative lens, a beam splitter, a mirror and polarized filters and allow for smartphone-based direct ophthalmoscopy^15^. Additionally eyes were imaged with conventional monoscopic fundus photography (CFP) (Visucam 500, Carl Zeiss Meditec, Jena, Germany), also dilated. The Galaxy S4 backside camera is equipped with a 12.8 megapixel CMOS (“complementary metal-oxide-semiconductor”) sensor and the Visucam 500 with a 5.0 megapixel CCD (“charge-coupled device”) sensor. The working distance for image acquisition was 10–30 mm for the Galaxy S4 with the D-Eye adapter and 40 mm for the Visucam 500. SBFP was performed by the same examiner, experienced in direct and indirect SBFP, in all patients (MWMW) in a darkened room. After 5 minutes of unsuccessful imaging attempts examination was aborted.

### Image analysis

**Figure 2:**
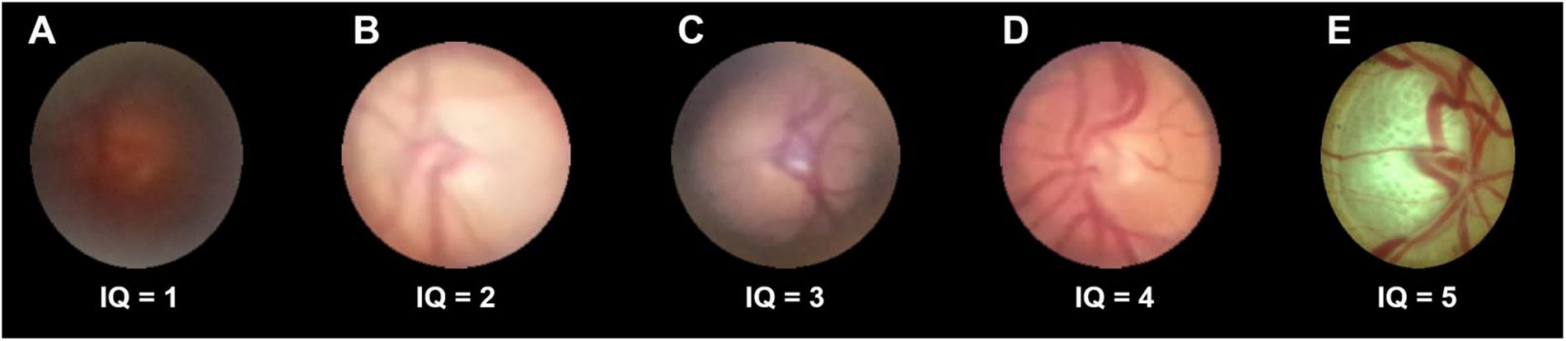
Image quality grading. Image quality (IQ) was graded on a 6-step-scale with these exemplary images for 0 = no fundus details visible, 1 = trail of major vessels visible (**A**), 2 = trail of medium size vessels visible (**B**), 3 = trail of some small vessels visible (**C**), 4 = trail of nearly all small vessels visible (**D**), 5 = even small vessels sharp (**E**).

**Figure 3:**
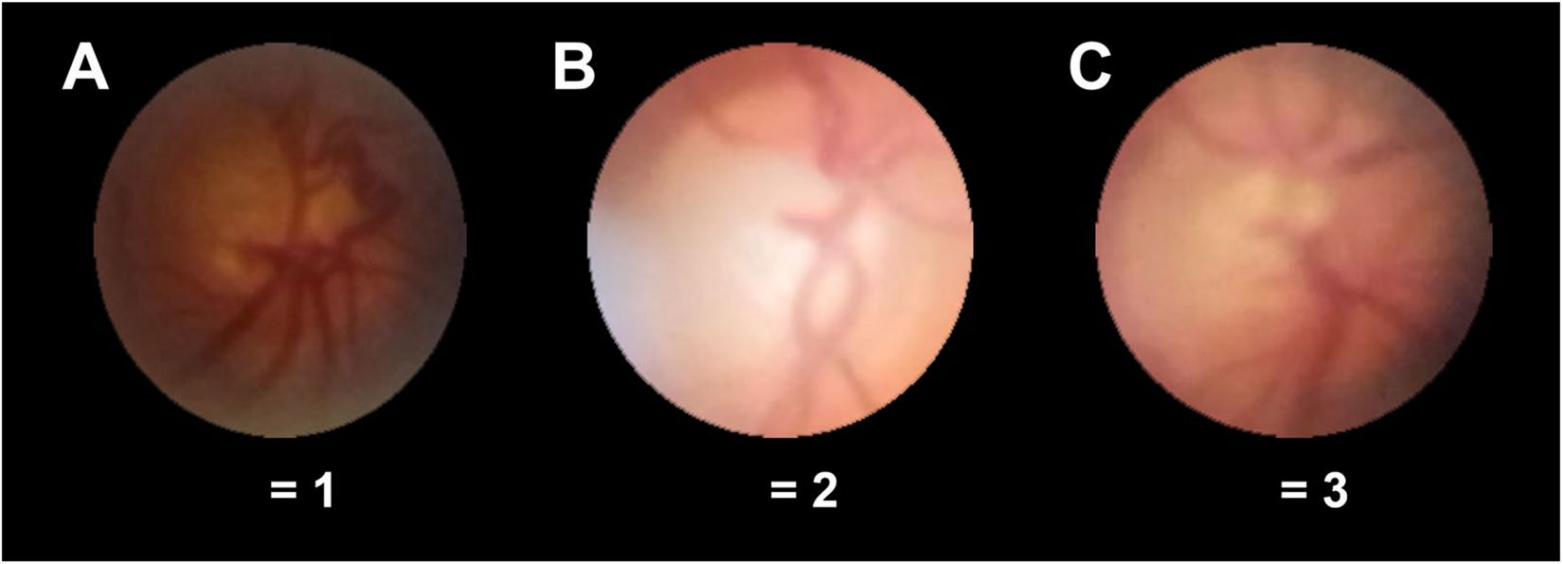
Optic disc rim visualization. Degree of optic disc rim visualization was graded on a 3-step-scale with these exemplary images for 1 = less than ¾ of the optic disc rim identifiable (**A**), 2 = at least ¾ of the optic disc rim identifiable (**B**, the lower left of the optic disc rim is overlaid by a reflex) and 3 = optic disc rim completely identifiable (**C**).

**Figure 4:**
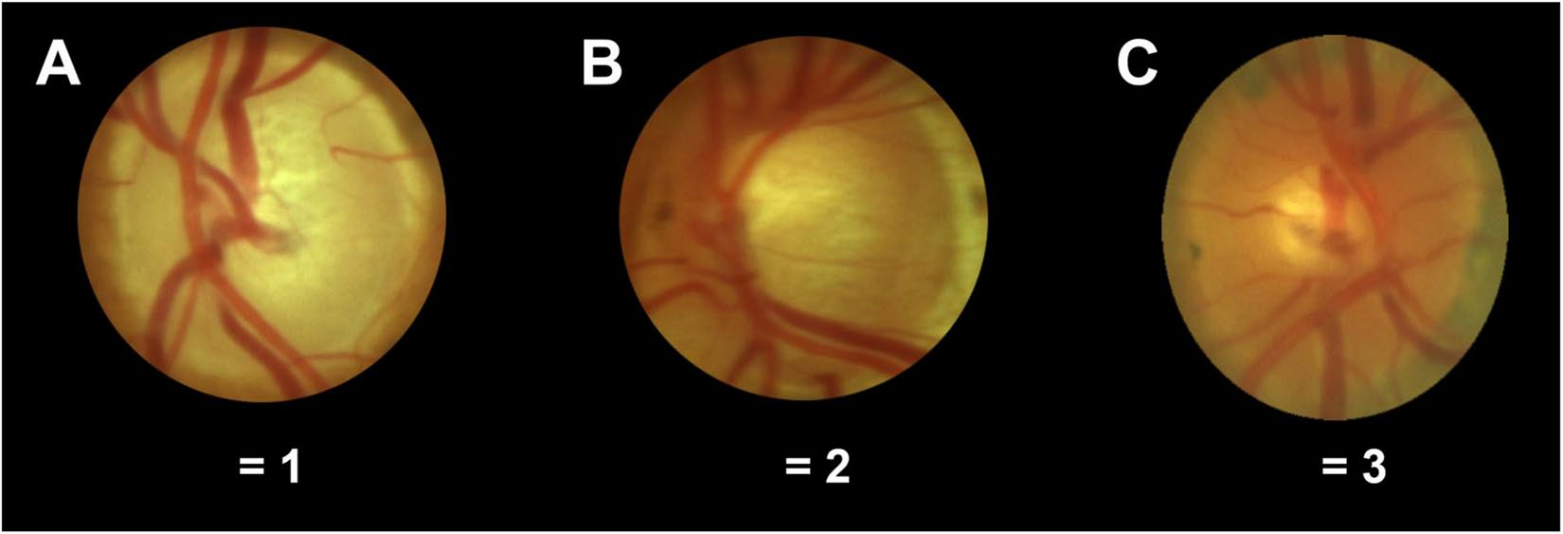
Optic disc pallor grading. Optic disc pallor was graded on a 3-step-scale with these exemplary images for 1 = pale (**A**), 2 = borderline (**B**) and 3 = vital (**C**).

For analysis only the central circular part of the image was cropped using Image J^17^ in order to mask for the status of dilation. All images were pseudonymized and analyzed by two masked graders (MWMW and CKB). Image quality was graded using a six-step-scale with exemplary images based on vessel visibility (Fig. 2). Optic disc rim visualization and degree of optic disc pallor were evaluated using a respective three-step-scale with exemplary images (Figs 3 and 4). VCDR was evaluated by measuring the total ONH height and the height of the superior and inferior neuroretinal rim with Image J, entering the results in an Excel table and vCDR was automatically calculated. Statistical analyses were performed with R (R: A Language and Environment for Statistical Computing, R Core Team, R Foundation for Statistical Computing, Vienna, Austria, 2016). Photographic vCDR assessment on CFP was compared with assessment from the medical records based on stereoscopic slit lamp biomicroscopy. Weighted kappa (for image quality, optic disc pallor and degree of optic disc rim visualization) and intraclass correlation (for vCDR) were calculated to assess inter-observer reliability.

### Data availability

The datasets generated during and analyzed during the current study are available from the corresponding author on reasonable request.

## Results

### Subject demographics and clinical characteristics

54 eyes (27 participants) were included in the study. All participants were imaged bilaterally. Characteristics of the sample are displayed in Table 1.

**Table 1:** Demographics.

		Mean ± SD or n (%)
Age		70 ± 11.3
Age range		35–89
vCDR		0.76 ± 0.14
vCDR range		0.42–0.96
Lens status	phakic	21 (39%)
	pseudophakic	33 (61%)
Sex	Male	26 (48%)
	Female	28 (52%)

SD = standard deviation.

### Evaluation of image quality and optic disc rim visualization

**Figure 5:**
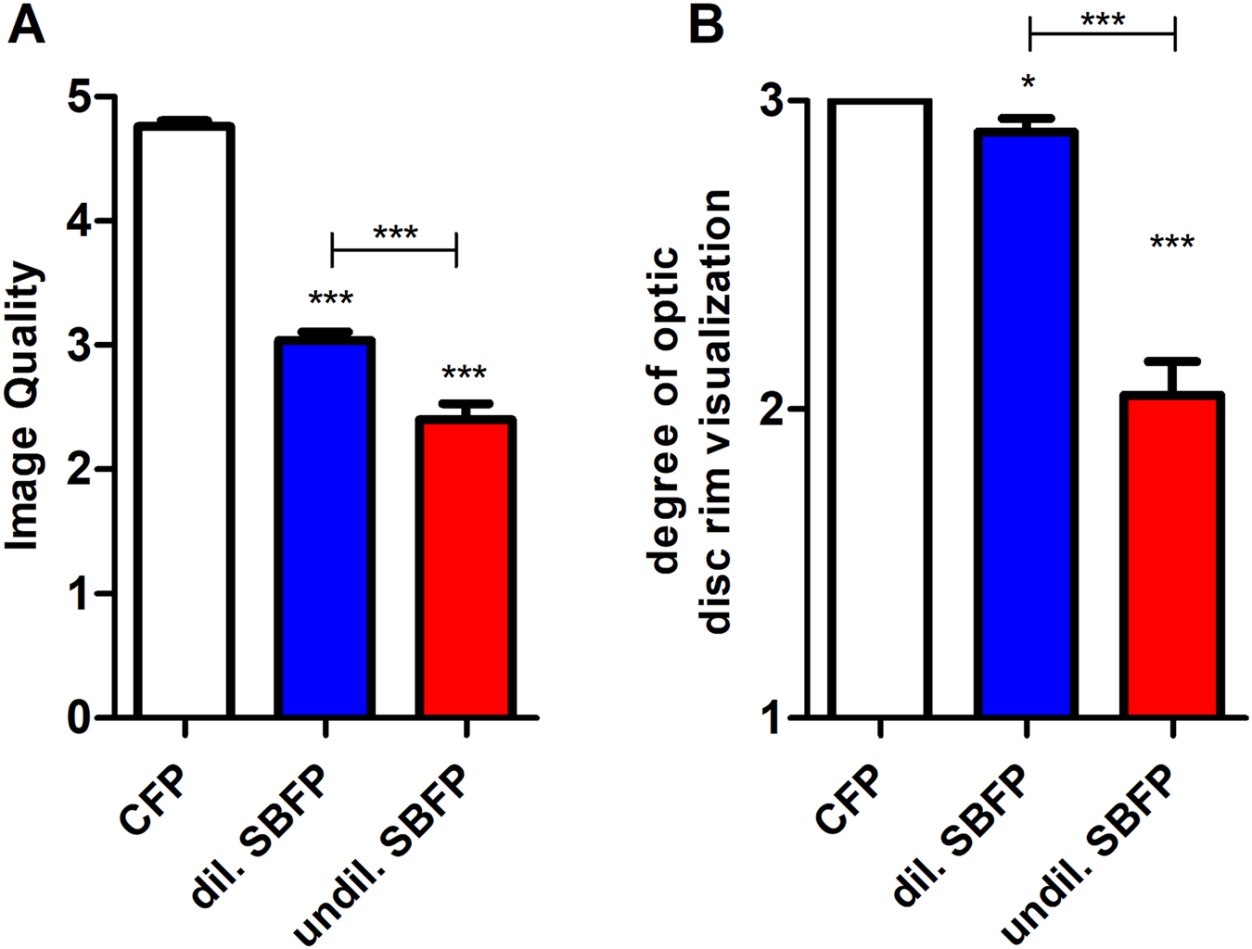
Evaluation of Image quality (**A**) and degree of optic disc rim visualization (**B**). Conventional fundus photography (CFP) and dilated (dil.) and undilated (undil.) smartphone-based fundus photography (SBFP) were compared. Error bars indicate standard error of the mean, paired t-test was used for analysis, asterisks indicate significant differences in regards to CFP if not otherwise indicated, *p < 0.05, ***p < 0.001.

SBFP was possible in 74% without dilation and in 98% following dilation. Reasons for failure of SBFP were small pupil diameter, media opacity or the combination of both, based on subjective evaluation during examination. Mean image quality ± standard deviation (SD) on CFP and on dilated and undilated SBFP was 4.8 ± 0.36, 3.0 ± 0.50 and 2.4 ± 0.94, respectively (p < 0.001, Fig. 5A, for parametric testing see Table 2). Weighted kappa in between graders for image quality and degree of optic disc rim visualization were 0.73 and 0.83, respectively.

**Table 2:** Parametric testing for the comparison of the different imaging modalities.

	Difference of the mean	p-value	95% Confidence interval
**Image quality**			
CFP vs. dilated SBFP	1.72	**<0**.**001**	1.56–1.85
CFP vs. undilated SBFP	2.36	**<0**.**001**	2.10–2.62
Dilated vs. undilated SBFP	0.64	**<0**.**001**	0.41–0.87
**Degree of optic disc rim visualization**			
CFP vs. dilated SBFP	0.10	**0**.**03**	0.01–0.19
CFP vs. undilated SBFP	0.95	**<0**.**001**	0.74–1.17
Dilated vs. undialted SBFP	0.85	**<0**.**001**	0.62–1.08
**Vertical Cup-to-disc evaluation**			
CFP vs. dilated SBFP	0.03	**<0**.**001**	0.02–0.05
CFP vs. undilated SBFP	0.08	**<0**.**001**	0.06–0.12
Dilated vs. undialted SBFP	0.05	**<0**.**001**	0.03–0.09
**Optic disc pallor evaluation**			
CFP vs. dilated SBFP	−0.02	0.82	−0.18–0.14
CFP vs. undilated SBFP	−0.20	**0**.**005**	−0.41– −0.08
Dilated vs. undialted SBFP	−0.18	**0**.**03**	−0.39– −0.02

CFP = conventional fundus photography; SBFP = Smartphone-based fundus photography.

Mean degree of optic disc rim visualization ± SD on CFP and on dilated and undilated SBFP was 3.0 ± 0, 2.9 ± 0.33 and 2.0 ± 0.79, respectively (Fig. 5B, for parametric testing see Table 2). Degree of optic disc rim visualization was nearly one step lower in undilated SBFP compared to dilated SBFP and CFP, whereas there was only a small difference between dilated SBFP and CFP.

### Evaluation of the optic nerve head

**Figure 6:**
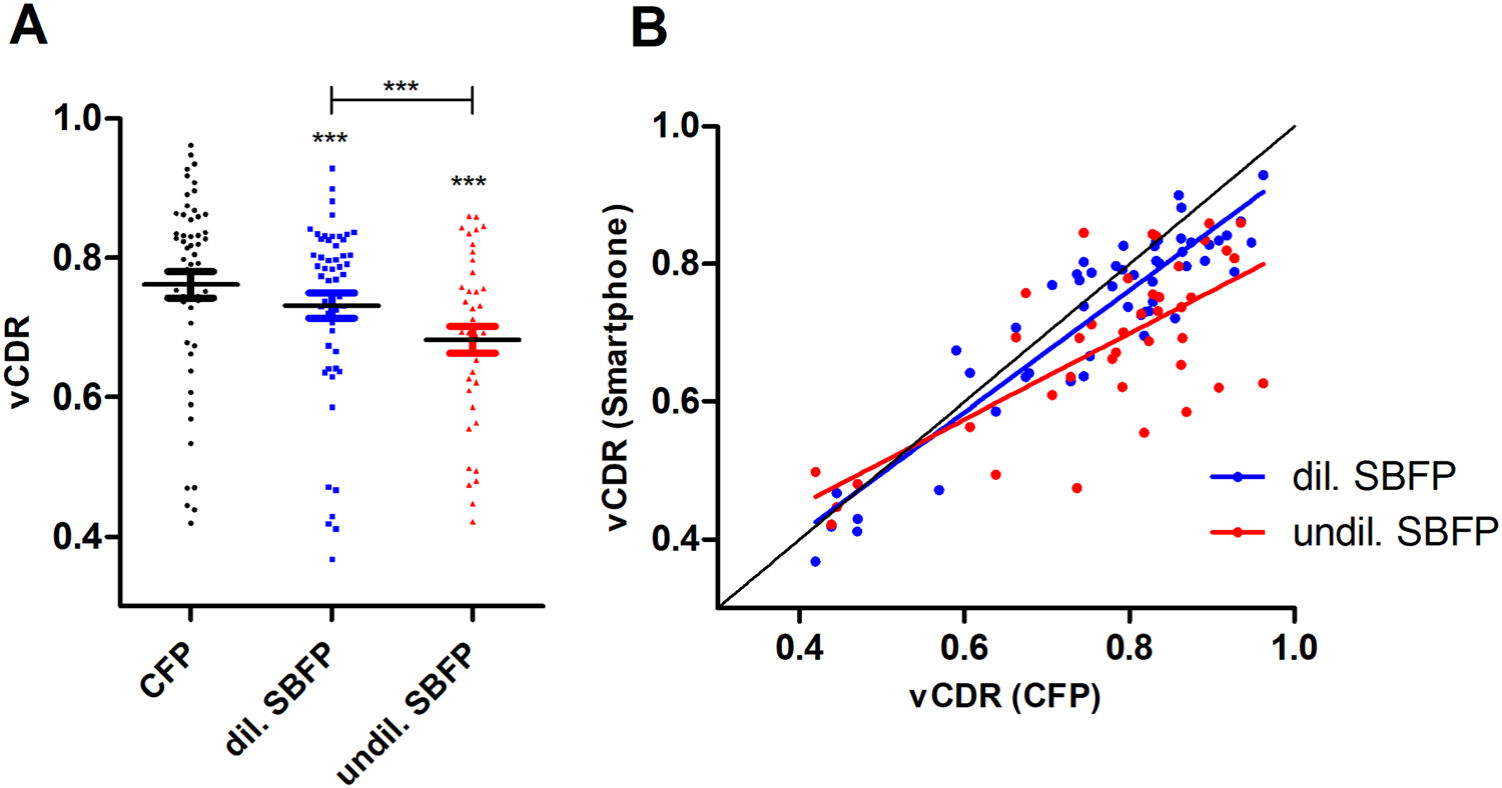
Evaluation of vertical Cup-to-disc ratio. Conventional fundus photography (CFP) and dilated (dil.) and undilated (undil.) smartphone-based fundus photography (SBFP) were compared. (**A**) Comparison of the individual devices and (**B**) correlation of vertical Cup-to-disc ratio (vCDR) measurement on dil. and undil. SBFP with vCDR-measurement on CFP. Error bars indicate standard error of the mean, paired t-test was used for analysis, asterisks indicate significant differences in regards to CFP if not otherwise indicated, ***p < 0.001.

Mean vCDR ± SD on CFP and on dilated and undilated SBFP was 0.76 ± 0.14, 0.73 ± 0.13 and 0.68 ± 0.12, respectively. vCDR was underestimated on both dilated and undilated SBFP, however underestimation was greater on undilated SBFP (Fig. 6A, for parametric testing see Table 2). Dilated SBFP correlated better with CFP (Pearson coefficient of correlation r = 0.91, p < 0.001) than undilated SBFP (r = 0.70, p < 0.001) (Fig. 6B). Intraclass correlation for vCDR between graders was 0.81. Photographic vCDR assessment on CFP correlated well with stereoscopic slit lamp biomicroscopy (Pearson coefficient of correlation r = 0.82, p < 0.001).

**Figure 7:**
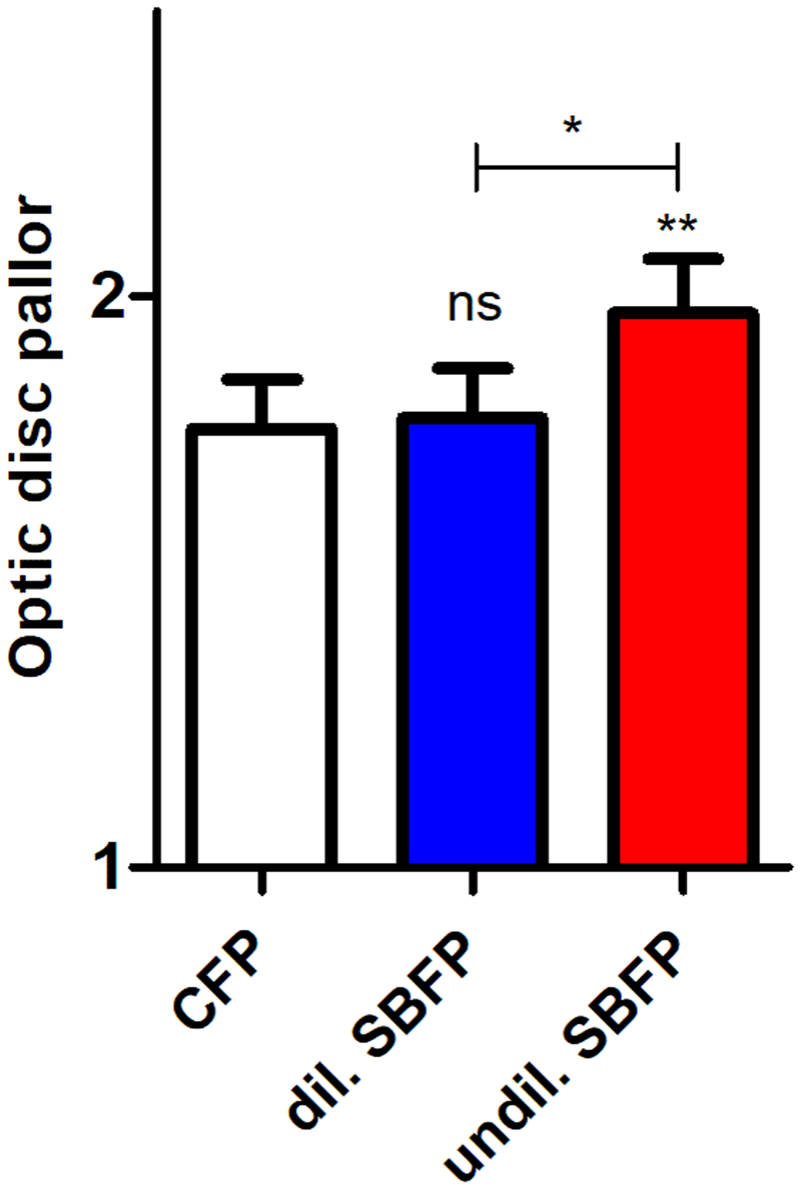
Evaluation of optic disc pallor. Conventional fundus photography (CFP) and dilated (dil.) and undilated (undil.) smartphone-based fundus photography (SBFP) were compared. Error bars indicate standard error of the mean, paired t-test was used for analysis, ns = not significant, *p < 0.05, **p < 0.01.

There was no difference in optic disc pallor evaluation in between CFP and dilated SBFP, however there was a bias towards evaluating optic nerve heads as less pale on undilated SBFP (Fig. 7, for parametric testing see Table 2). Mean optic disc pallor ± SD on CFP and on dilated and on undilated SBFP was 1.77 ± 0.63, 1.79 ± 0.64 and 1.97 ± 0.68, respectively. Weighted kappa in between graders for optic disc pallor was 0.38.

### Subgroup analysis phakic vs. pseudophakic eyes

Statistically significant differences concordant to the overall analysis for image quality and degree of optic rim visualization and differences concordant, however not statistically significant, for vCDR evaluation were present (see Table 3). While a concordant, yet not statistically significant difference was evident for optic disc pallor evaluation in phakic eyes, difference was nearly zero for pseudophakic eyes (see Table 3).

**Table 3:** Comparison of dilated and undilated smartphone-based fundus photography in phakic vs. pseudophakic eyes.

	Mean ± SD (dil. SBFP)	Mean ± SD (undil. SBFP)	Difference of the mean	p-value	95% Confidence interval
**Image quality**					
Phakic	3.38 ± 0.74	2.57 ± 1.40	0.81	**0**.**026**	0.10–1.51
Pseudophakic	3.39 ± 0.75	2.79 ± 0.93	0.61	**0**.**005**	0.19–1.02
**Degree of optic disc rim visualization**					
Phakic	3 ± 0	2.33 ± 0.97	0.67	**0**.**005**	0.23–1.11
Pseudophakic	2.91 ± 0.29	2.09 ± 0.77	0.82	**<0**.**001**	0.53–1.11
**Vertical Cup-to-disc evaluation**					
Phakic	0.77 ± 0.14	0.71 ± 0.15	0.06	0.20	−0.04–0.16
Pseudophakic	0.75 ± 0.13	0.70 ± 0.12	0.05	0.16	−0.02–0.12
**Optic disc pallor evaluation**					
Phakic	1.76 ± 0.70	2.05 ± 0.78	− 0.29	0.22	−0.77–0.19
Pseudophakic	1.55 ± 0.71	1.53 ± 0.72	0.01	0.94	−0.34–0.37

CFP = conventional fundus photography; dil. = dilated; SBFP = Smartphone-based fundus photography; SD = standard deviation; undil. = undilated.

## Discussion

This is the first study comparing dilated and undilated SBFP. Overall SBFP success rate, image quality and optic disc rim visualization were better following dilation for the tested adapter and vCDR evaluation on dilated SBFP correlates well with vCDR evaluation on CFP, whereas evaluation on undilated SBFP potentially underestimated vCDR. Similarly, disc pallor was underestimated on undilated SBFP. This highlights the need for dilation in order to ascertain good quality ONH evaluation using SBFP. However, CFP still outperformed SBFP.

The findings further support existing data showing that ONH evaluation is possible with SBFP^11,12^. Possible explanations for reduced image quality and impaired optic disc rim visualization are constrained optics in case of undilated pupils. Only if the illumination’s and the camera’s light beam are perfectly coaxial and centered in the pupil sufficient image quality can be achieved. Especially in very small pupils this is hard to achieve and even if it is achieved the small pupil diameter might lead to backscatter from the pupil margins leading to increased blurring of the image. As a result from this the blurred neuroretinal rim appears wider and kinking of small vessels into the excavation of the disc cannot be recognized clearly anymore or even not at all. Hence, vCDR and vitality of the ONH might be misinterpreted. Additionally, when comparing CFP with SBFP it is important to take into account that the quality of the optics and the optical setup of smartphones equipped with adapters for SBFP is not as good and well adapted to fundus imaging as are professional fundus cameras. This is most likely the reason why image quality is lower in dilated SBFP than in CFP, although the image resolution achieved with the Galaxy S is much higher than with the Visucam 500 (13 vs. 5 megapixels).

Our subgroup analysis comparing phakic vs. pseudophakic eyes indicated that the difference in optic disc pallor evaluation between dilated and undilated SBFP might be greater in phakic eyes. However this assumption cannot be validated, as we did not compare the same eyes before and after cataract surgery.

As an implication of the results reported herein ONH evaluation with SBFP should be performed following pupil dilation. Nevertheless in cases where dilation might not be possible, e.g. in an outreach screening camp with very limited or no ophthalmic resources, ONH evaluation with SBFP adapters like the D-Eye, which allow for undilated ONH evaluation, represent a possible alternative. Further technical improvements and developments such as utilization of higher quality optical systems are warranted.

The D-Eye is not the only adapter allowing for SBFP. Both the PEEK vision^12^ and the Welch Allyn iExaminer^16^ are two possible alternatives, with only the latter allowing for undilated SBFP. As no comparison between the different adapters has been done so far further studies are needed to evaluate and compare the available smartphone adapters. Additionally experience levels of different examiners should be taken into account, as this can affect image quality^18^.

The strengths of our study are a direct comparison within the same patient, a patient sample with a wide range of age and vCDR, two masked graders and a comprehensive evaluation of imaging quality factors (image quality and degree of optic disc rim visualization) and ONH evaluation (vCDR and optic disc pallor). The limitations of our study are the small sample size, no comparison between different smartphone adapters, the high number of advanced glaucoma cases and that we did not assess the pupil size, intra-patient repeatability and intra-observer reliability of the examination. Furthermore the use of monoscopic imaging bears the risk of a bias towards lower vCDR measurements and reduced inter-observer agreement^19^.

Smartphone-based fundus photography for ONH evaluation is promising and dilation further increases its quality. It may become a feasible alternative to conventional cameras in the future, allowing for a novel approach to tele-ophthalmology. However, conventional CFP following dilation still outperforms SBFP and further development of smartphone adapters for fundus photography is warranted.
